# Neutrophil-activating protein in *Bacillus* spores inhibits casein allergy via TLR2 signaling

**DOI:** 10.3389/fimmu.2024.1428079

**Published:** 2024-11-05

**Authors:** Zhuwei Liang, Chao Zhang, Xiaoyu Liu, Kaiyue Yang, Zhile Xiong, Bingshao Liang, Jialiang Mai, Xiaojun Xiao, Jie Liu, Pingchang Yang, Damo Xu, Zhenwen Zhou

**Affiliations:** ^1^ Clinical Laboratory, Longgang Maternity and Child Institute of Shantou University Medical College (Longgang District Maternity & Child Healthcare Hospital of Shenzhen City), Shenzhen, Guangdong, China; ^2^ Clinical Laboratory, Guangdong Provincial Second Hospital of Traditional Chinese Medicine (Guangdong Provincial Engineering Technology Research Institute of Traditional Chinese Medicine, Guangdong Provincial Key Laboratory of Research and Development in Traditional Chinese Medicine), Guangzhou, Guangdong, China; ^3^ The State Key Laboratory of Respiratory Disease for Allergy, Shenzhen Key Laboratory of Allergy & Immunology, Shenzhen University School of Medicine, Shenzhen, Guangdong, China; ^4^ National Clinical Laboratory on Tuberculosis, Beijing Key Laboratory for Drug-Resistant Tuberculosis Research, Beijing Chest Hospital, Capital Medical University, Beijing Tuberculosis and Thoracic Tumor Institute, Beijing, China; ^5^ Clinical Laboratory, Guangzhou Women and Children’s Medical Center, Guangzhou Medical University, Guangzhou, Guangdong, China; ^6^ Clinical Laboratory, Foshan Maternity and Child Health Hospital, Foshan, Guangdong, China; ^7^ Department of Respiratory & Allergy, Third Affiliated Hospital of Shenzhen University, Shenzhen, Guangdong, China

**Keywords:** *B. subtilis* spores, casein allergy, neutrophil-activating protein, toll-like receptor 2, oral immunotherapy

## Abstract

**Background:**

Milk allergy commonly occurs in children, mainly caused by bovine-derived casein (CAS) protein. Neutrophil-activating protein (NAP) of *Helicobacter pylori* plays an immunomodulatory role with potential to suppress Th2-type immune responses. *Bacillus subtilis* (*B. subtilis*) spores are commonly used as oral vectors for drug delivery.

**Objective:**

To investigate whether recombinantly expressed NAP on *B. subtilis* spores could be an effective treatment for CAS allergy in mouse.

**Methods:**

After CAS sensitization, mice were orally administered *B. subtilis* spores expressing recombinant NAP for 6 weeks. Allergic symptoms and parameters were evaluated after CAS challenge oral gavage, including allergic inflammation, splenic cytokines, and serum-specific antibodies. Protein levels of Toll-like receptor 2 (TLR2) and c-JUN in the jejunum tissue were measured by western blot. Bone marrow-derived macrophages (BMDMs) were stimulated with inactivated NAP spores to measure the influence on cytokine profiles *in vitro*.

**Results:**

NAP recombinant spore treatment significantly reduced allergic symptoms and intestinal inflammation. Interleukin-12 and interferon-gamma levels increased, whereas serum CAS-specific IgG1 and IgE levels decreased. TLR2 and c-JUN expression levels were elevated in the jejunal tissue. Inactivated NAP spores polarized BMDMs to the M1 phenotype and enhanced cytokine expression, which were inhibited by a TLR2 neutralizing antibody.

**Conclusion:**

NAP offers a new strategy in the treatment of CAS allergy by inhibiting the Th2 response, while eliciting macrophages to promote Th1 immune responses.

## Graphical Abstract

Oral treatment of recombinant Bacillus subtilis spore was found effective against casein allergy, which delivered neutrophil-activating protein (NAP) of Helicobacter pylori. We explored that recombinant CotC-NAP spores could elicit macrophages M1 polarization through TLR2-dependent signaling pathway, which activate phosphorylation of NF-κB and c-JUN, further release cytokines like interleukin-12 and interferon-gamma to rectify the balance of Th1/Th2 and regulate the level of Tregs.

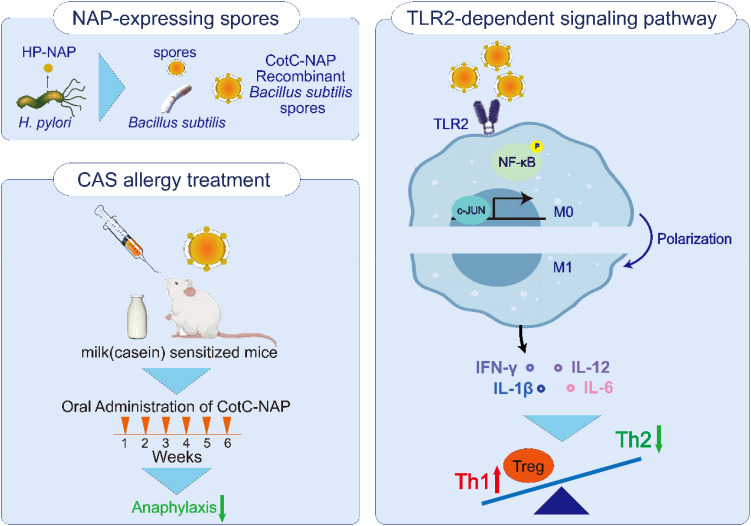

## Introduction

The prevalence of food allergy is on the rise, representing a major global public health issue (1). Milk allergy is common and most likely to occur in children (2), which poses challenges in obtaining the component nutrients. Casein (CAS), accounting for approximately 80% of bovine-derived milk proteins (3), is the main allergenic protein of milk. A lower level of serum CAS-specific IgE is associated with greater milk tolerance in humans (4–6). Although allergen immunotherapy has achieved certain efficacy in the treatment of food allergy, recurrent allergic reactions are common and the overall safety profile is unsatisfactory, including side effects such as local or serious systemic reactions, which hinder treatment adherence (7). Therefore, it is essential to develop an effective and safe treatment against food allergens such as CAS.

A Th1/Th2 immune imbalance is considered the main immune mechanism underlying food allergy. Overactivation of the Th2 immune response results in the production of a large amount of antigen-specific IgE antibody, thereby releasing allergic mediators and an increase of eosinophil infiltration in the tissue (8). Interferon (IFN)-γ released from Th1 immune cells can inhibit the immune response of Th2 cells (9), thereby suppressing Th2-mediated allergic reactions. Toll-like receptors (TLRs) play critical roles in immune defense by identifying conserved structures of multiple microbial products to initiate and regulate specific immune responses (10). TLR2 recognizes the largest variety of pathogens and has the widest expression range among the TLRs, representing an important link between natural and regulatory immunity (11–13). TLR2 can activate nuclear factor (NF)-κB to produce interleukin (IL)-12 through the MyD88-dependent pathway, thereby inducing the differentiation of initial T cells toward Th1 cells (14). Thus, selectively activating TLR2, promoting Th1 immune responses may provide a more effective therapeutic mechanism for CAS allergy.

The incidence of asthma in children is negatively correlated with the colonization rate of *Helicobacter pylori* (HP) and the infection rate of HP is inversely proportional to the incidence of allergic diseases (15, 16). HP expresses neutrophil-activating protein (NAP), which has emerged as a therapeutic target and a candidate protective antigen for vaccine development against HP infection (17). NAP has recently been shown to act as a powerful immunomodulator capable of inducing the production of endogenous IL-12 (18). These findings suggest that NAP can trigger a biased Th1-type immune response and generate anti-Th2 activity. Therefore, we hypothesized that NAP could also inhibit the CAS-induced allergic response. In an asthma model, NAP inhibited the Th2 immune response, but did not significantly suppress asthma after *Tlr2* knockdown (19). However, the molecular mechanism driven by TLR2 to mediate the effects of NAP on the release of cytokines from food allergens on intestinal epithelial cells and the subsequent downstream immune response in intestinal food allergy remain unclear.

*B. subtilis* is widely present in the natural environment and in the human microbiota, which is used as a probiotic and food additive (20). *B. subtilis* produces spores with excellent stability that withstand harsh environments. Therefore, most spores can survive after passing through the gastrointestinal tract, demonstrating good application prospects as an oral vaccine carrier (21, 22). Moreover, the coat protein CotC of *B. subtilis* spores shows ductility, enabling coupling and fusing of foreign genes that can be expressed together on the surface of the spores without affecting their formation and germination (23). Using CotC as an anchor protein, we successfully fused and expressed NAP on the surface of *B. subtilis* spores (24–27). We also found that NAP recombinant spores reduced asthma and peanut allergy; however, the in-depth molecular mechanism remains to be elucidated (27, 28).

In this study, we evaluated the effectiveness of oral gavage of NAP recombinant *B. subtilis* spores to mice with CAS-induced IgE-mediated type I hypersensitivity. To uncover the potential mechanism, we explored whether NAP could activate TLR2 signaling and thus inhibit the allergic inflammation induced by CAS.

## Methods

### Ethics approval and consent to participate

This study was approved by the Ethics Committee of Guangzhou Women and Children’s Medical Center (registration no. 108B00). No human or related specimens were used in this study, and we declare that our study complies with the Declaration of Helsinki.

### Immunofluorescence

The recombinant plasmids pUS186-CotC and pUS186-CotC-NAP in *B. subtilis* WB600 were previously constructed in our laboratory, which can be viewed in our reported articles (25–27). We used our previously constructed specific rat anti-NAP serum as the primary antibody for immunofluorescence (27). The recombinant spores were resuspended in buffer (0.01% glutaraldehyde, 2.4% paraformaldehyde, and 30 mm NaPO_4_, pH 7.4), fixed at 25°C for 15 min, and placed on ice for 35 min. The spores were washed in phosphate-buffered saline (PBS) three times and resuspended with 125 µl PBS. Lysozyme (4 mg/ml) was added and then 2.5 µl of the suspension was applied to a slide to dry naturally. The slide was blocked with 2% bovine serum albumin (BSA)-PBS at 25°C for 1 h. After washing twice with PBS, the slides were incubated with rat NAP antibody positive serum (1:50) at 25°C for 2 h. After washing with PBS three times, the slides were incubated with anti-rat-IgG (Alexa Fluor 488) secondary antibody (1:200; Abcam, UK) at 25°C in the dark for 1 h. The slides were washed with PBS five times in the dark, dried, sealed with anti-fluorescence quenching sealing agent. Using bright field view and Alexa Fluor 488 channel under a 400x fluorescence microscope(Nikon) to confirm the recombinant spores.

### Sensitization, treatment, and challenge of mice

Female BALB/c mice aged 4–5 weeks (Guangdong Experimental Animal Center, China) were housed in a specific pathogen-free environment at Shenzhen University (Shenzhen, China). The implementation of this study protocol was approved by the Shenzhen University Animal Care and Use Committee (No. 201903600). All methods used in the study were carried out in accordance with relevant guidelines and regulations.

**Figure 1:**
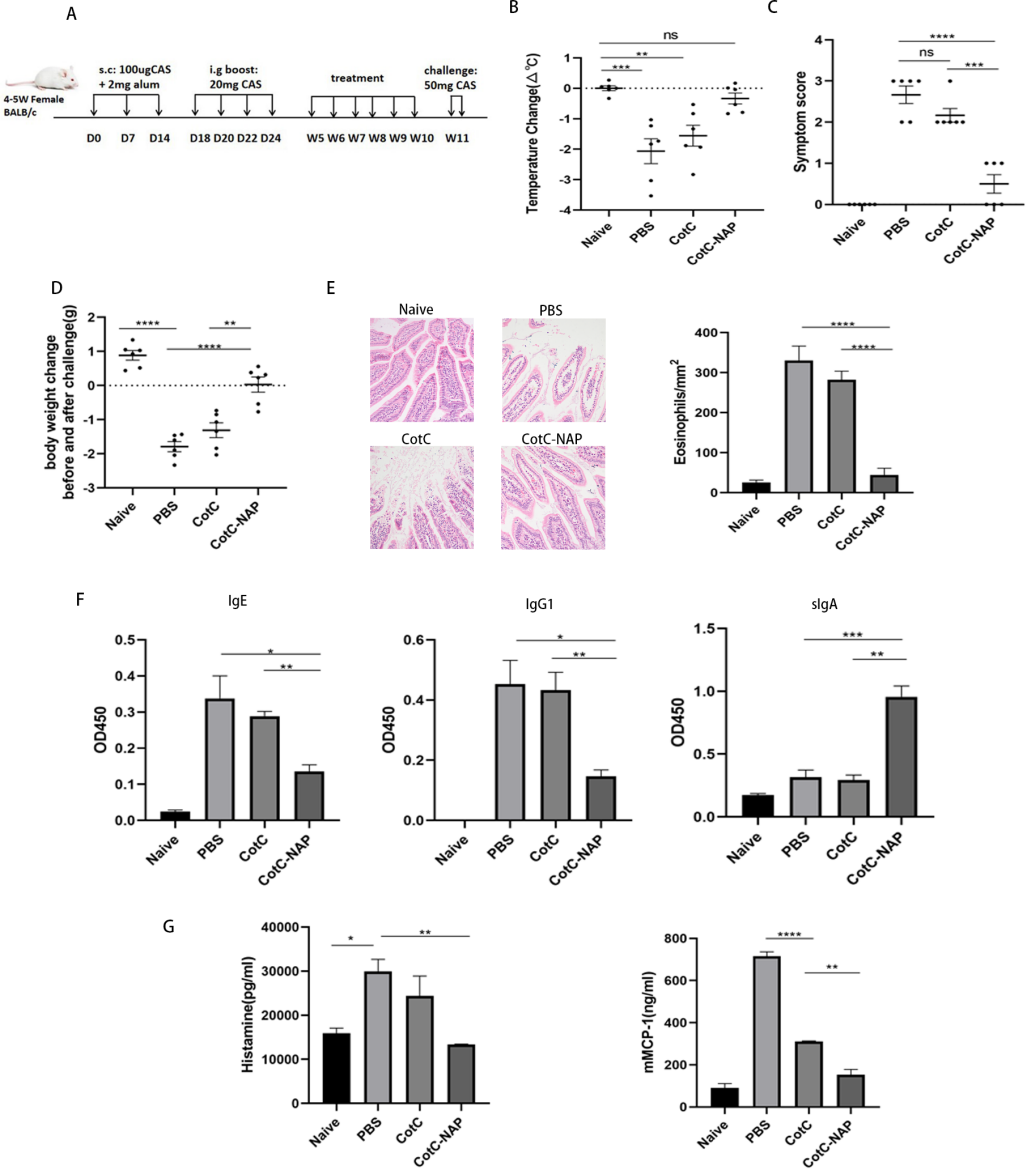
Recombinant spores expressing NAP reduce CAS-induced food allergy in mice. **(A)** Experimental protocol for establishment of the CAS allergy mouse model and immunotherapy with recombinant Bacillus subtilis. Mice in all groups except for the naïve group were sensitized with 100 μg CAS and 2 mg alum by subcutaneous injection on days 0, 7, and 14, and boost-sensitized with 20 mg CAS by gavage on days 18, 20, 22, and 24. Mice were gavaged with corresponding recombinant spores (1.0 × 10^9^) or the same volume of PBS for 6 weeks. Mice were challenged twice by gavage with 50 mg CAS at 48-h intervals at week 11. D: Day, W: Week. **(B)** Change in anal temperature after the second challenge. **(C)** Thirty minutes after the second challenge, the allergic symptoms of mice were observed and scored. **(D)** Difference in weight before and after the second challenge. **(E)** Histological assessment of paraffin-embedded sections of jejunal tissues stained with hematoxylin and eosin observed under a microscope at 200× magnification; 20 fields were randomly selected to count eosinophils with 400× magnification. **(F)** ELISA for CAS-IgG1, IgE, and IgG2a in the serum and CAS-sIgA in the feces. **(G)** Detection of mast cell degranulation level, including histamine and mMCP-1. Data are presented as mean ± SEM (n = 6/group). *P < 0.05, **P < 0.01, ***P < 0.001, ****P < 0.0001.

24 mice were randomly divided into 4 groups: naïve, PBS, CotC spores and CotC-NAP spores groups. As shown in **Figure 1A**, on days 0, 7, and 14, mice received subcutaneous injection of 100 µl CAS sensitization solution (2 mg/ml CAS; Sigma, St. Louis, MO, USA) and an equal volume of aluminum agent (ThermoFisher Scientific, Waltham, MA, USA). The naïve group mice did not receive any treatment, and came from the same litter as other mice. As a boost, on days 18, 20, 22, and 24, mice oral gavaged with 200 µl CAS challenge solution [100 mg/ml CAS with 3.3% vodka (Belalco, Russia) and 1.5% sodium bicarbonate (Aladdin, Shanghai, China)]. On the Day 15, the serum level of CAS-specific IgE was measured before spore treatment to confirm successful model establishment. From the 5^th^ to 10^th^ week, mice oral gavaged with corresponding recombinant spores (1.0 × 10^9^) or sterile PBS. At week 11, all mice were challenge twice by gavage with 50mg CAS challenge solution at a 48-h interval following overnight fast.

### Signs of hypersensitivity

Allergic symptoms were observed 30 min after the last challenge. Symptoms were scored according to previously described allergy scoring criteria (29) on a scale of 0–5 as follows: 0, no symptom; 1, scratching the ears and nose; 2, diarrhea, erect hair, swelling around the eyes and ears, shortness of breath, and inactivity; 3, dyspnea, wheezing, cyanosis; 4, immobility, muscle contraction, and cramps; 5, death, shock. The anal temperature of the mice was measured by murine anal thermometer 30 min before and after challenge.

### Histopathological examination of the jejunum

After sacrificing the mice challenged with CAS, cut off the jejunal tissue and cut into 1-cm sections, and fixed with 4% formalin for 24 h. After dehydrating with alcohol, the tissue was embedded with paraffin, cut into 4-µm-thick sections, fixed on slides, and stained with hematoxylin and eosin (Solarbio) for histopathological analysis.

### Detection of CAS-specific serum and intestinal mucosal antibodies

30 min after challenging the mice, we collected serum and feces to analyze the levels of CAS-specific IgE and IgG1 in serum and CAS-specific sIgA in feces by ELISA as previously described (28).

### Enzyme-linked immunosorbent assay

After sacrificing the mice challenged with CAS, plasma and serum were collected for the detection of histamine and mouse mast cell protease-1 (mMCP-1) with respective ELISA kits from Mlbio (Shanghai, China) and Invitrogen (USA) according to the manufacturer instructions.

### Splenocyte isolation, immune cell quantification, and cytokine production

After sacrificing the mice challenged with CAS, the lymphocytes of spleen were isolated and cultured with RPMI 1640 complete medium. 1 × 10^6^ cells were stained with fluorescein thiocyanate-CD4, allophycocyanin (APC)-CD25, APC-IL4 antibody, phycoerythrin (PE)-FOXP3, or PE-IFN-γ antibody (Biolegend, USA) and analyzed on a Beckmann flow cytometer. Another 1 × 10^6^ cells were cultured in 1 ml RPMI 1640 complete medium with 50 ng phorbol 12-myristate 13-acetate (Solarbio), 1 µg ionomycin (Sigma), and 40 µg CAS (filtered and bacteria removed) at 37°C with 5% CO_2_ for 72 h. Relevant cytokines were detected in the supernatant using corresponding ELISA kits (4Abio; Beijing, China) according to the manufacturer instructions.

### mRNA and protein expression in the jejunum

RNA was extracted from the jejunum tissue sections and synthesized to cDNA by RNA Extraction Kit (Tiangen, Beijing, China) and Superscript II reverse transcription kit (TransGen, Beijing, China), respectively. Quantitative polymerase chain reaction was performed with SYBR Green qPCR Master Mix (DBI Bioscience, Shanghai, China). Expression levels were quantified with the 2^−ΔΔCT^ method as fold change relative to levels of the untreated control. *Gapdh* served as the internal reference gene. Sangon Biotech (Shanghai, China) designed and synthesized the primer sequences (**Table 1**).

**Table 1:** Primer sequence for RT-qPCR.

Gene	Forward Primer	Reverse Primer
GAPDH	TGTAGACCATGTAGTTGAGGTCA	AGGTCGGTGTGAACGGATTTG
Foxp3	CCCAGGAAAGACAGCAACCTT	TTCTCACAACCAGGCCACTTG
IFN-γ	GAGCCAGATTATCTCTTTCTACC	GTTGTTGACCTCAAACTTGG
IL-4	TACCAGGAGCCATATCCACGGATG	TGTGGTGTTCTTCGTTGCTGTGAG
IL-12	GACCTGTTTACCACTGGAACTA	GATCTGCTGATGGTTGTGATTC
IL-6	CTCCCAACAGACCTGTCTATAC	CCATTGCACAACTCTTTTCTCA
IL-1β	CACTACAGGCTCCGAGATGAACAAC	TGTCGTTGCTTGGTTCTCCTTGTAC

To detect protein levels, 100 mg jejunum samples were ground to a homogenate on ice with 800 µl RIPA tissue lysate containing 1 mM phenylmethylsulfonyl fluoride (Solarbio). Protein (30 µg) was added to the wells of a 12.5% SDS-PAGE gel containing 4× loading buffer (Beyotime) for separation, followed by transfer to a methanol-pretreated polyvinylidene fluoride membrane (Merck Millipore). Blocked it with 5% skimmed milk and incubated overnight with anti-β-actin [3700S, Cell Signaling Technology (CST), USA], anti-TLR2 (13744S, CST), anti-c-JUN (9165S, CST). Then incubated with HRP-conjugated secondary antibody (CST). The bands were detected using the UVIEC western blotting analysis system. Finally, the relative protein levels were determined by the density analysis method of Image J software.

### Culture and stimulation of bone marrow-derived macrophages

The recombinant *B. subtilis* spores were completely inactivated by heat treatment at 131°C for 30 min. The cells were induced by granulocyte/macrophage colony-stimulating factor (Sino Biological, Beijing, China) for 7 days to differentiate into macrophages. The cells were inoculated onto a 6-well plate at 1 × 10^6^ cells/well, and 50 µl inactivated spores (multiplicity of infection = 100) was added to each well; 50 µl RPMI 1640 complete medium was added as the negative control. After incubation at 37°C for 48 h, the cells were collected for flow cytometry to detect F4/80^+^CD86^+^ M1-type macrophages and for western blotting to detect the expression of TLR2, p-NFκB, NFκB, and c-JUN as described above.

To detect the role of TLRs in the immune response, 20 μg/ml neutralizing antibodies against TLR2 or TLR4 (14-9021-82, 558293, Invitrogen) were added to the cells and incubated at 37°C for 30 min; non-treated cells served as the control. Subsequently, 50 μl of the spores were added and incubated for 48 h. Three parallel experiments per well were established.

### Data analysis

The data are presented as mean ± standard error of the mean. Samples showing a normal distribution were analyzed for statistical significance by an independent t-test or analysis of variance, whereas samples with a non-normal distribution were analyzed by the Wilcoxon rank-sum test. All statistical analyses were performed in GraphPad Prism version 8.0.2 software and *P* < 0.05 was considered to indicate statistical significance.

## Results

### Identification and intestinal colonization of recombinant spores

**Figure 2:**
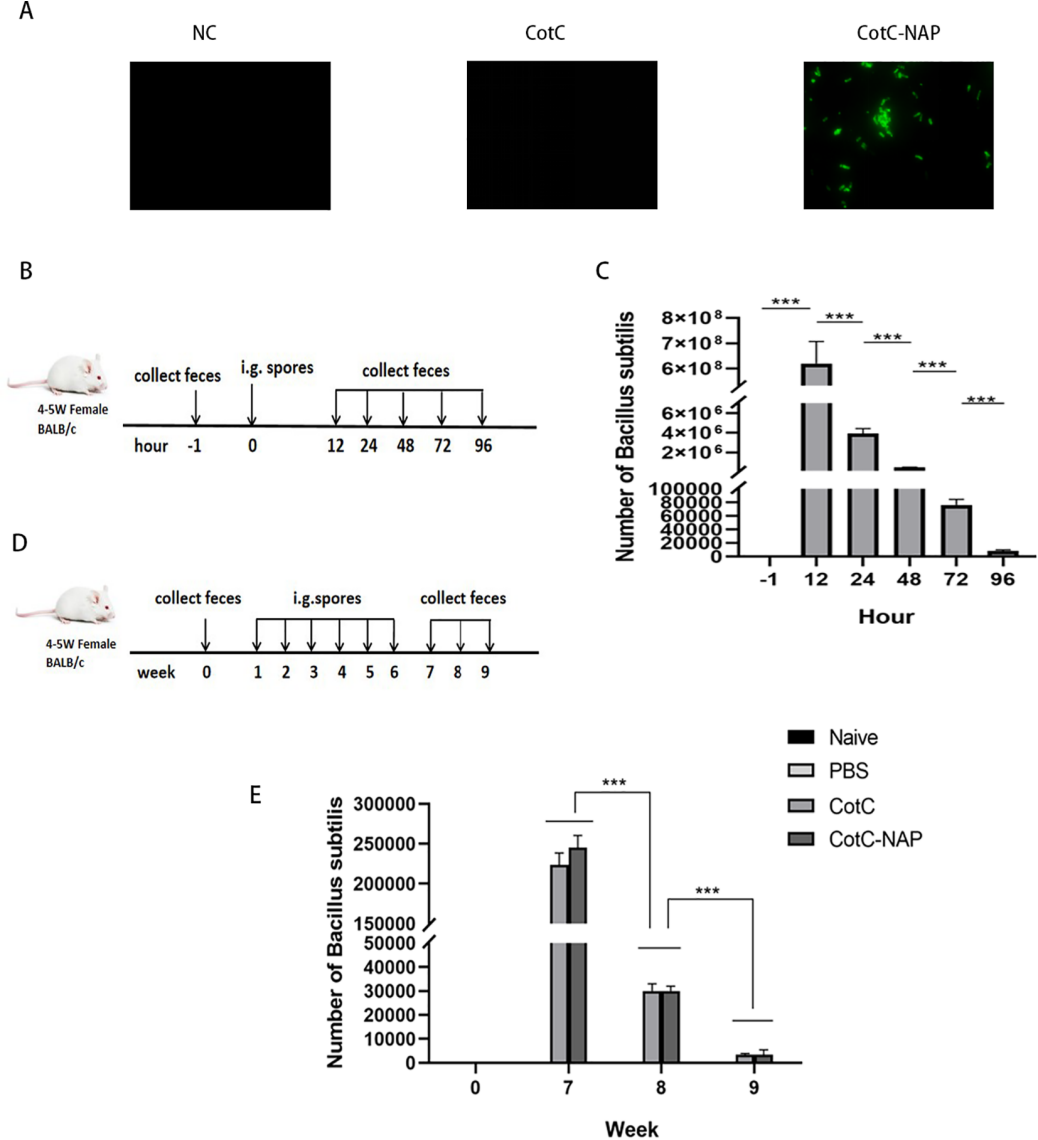
Identification and intestinal colonization of recombinant spores. **(A)** Immunofluorescent detection of recombinant spores (400×). Anti-NAP serum and the Alexa fluor488 fluorescent-conjugated anti-rat IgG antibody were incubated in sequence to observe the spores with a fluorescence microscope in the dark. **(B)** Protocol for detecting the number of spores colonizing the intestine after a single dose of CotC recombinant spores via gavage (1.0 × 109). Feces were collected at –1, 12, 24, 48, 72, and 96 h after gavage. **(C)** Feces (0.1 g) were resuspended with 1 ml saline and 10 μl suspensions were inoculated on KaNa+LB plates to count colonies and calculate the number of **(B)** subtilis spores per gram of feces. **(D)** Protocol for detecting the timing of spore colonization after 6 weeks of continuous gavage; gavage was performed every 48 h for 6 weeks. **(E)** Colony count of **(B)** subtilis spores per gram of feces in all groups. Data are presented as mean ± SEM (n = 6/group). ***P < 0.001.

No green fluorescence (Alexa fluor488 channel) was detected on the surface of NC group and CotC spores, whereas visible fluorescence was detected on the surface of spores expressing NAP (**Figure 2A**) through connecting with the capsid protein CotC. No colony growth in the feces of the PBS group was detected on the KaNa^+^LB plate at any time point. Numerous colonies were observed at 12, 24, 48, 72, and 96 h after gavage. They had the same morphology with a rough surface and opaque, light yellow color. Mass spectrometry (Bruker Daltonik MALDI Biotyper) confirmed that these colonies were *B. subtilis*. The number of spores in the feces increased significantly 12 h after gavage compared with that before gavage and were maintained for 96 h (**Figures 2B, C**, *P* < 0.001). And compared with the naïve and PBS groups, the number of spores in the experimental groups was the highest at week 7 and was maintained for 3 weeks (**Figures 2D, E**).

### Recombinant spores expressing NAP reduce CAS-induced food allergy in mice

The anal temperature of mice in PBS and CotC groups decreased significantly compared with that of mice in the untreated naïve group (**Figure 1B**, *P* < 0.01); however, there was no change in NAP group. The allergy symptom score is shown in the **Figure 1C**. Compared with that of the naïve group, the body weight decreased significantly after challenge in the PBS and CotC groups. However, NAP group has shown significant improvement (**Figure 1D**). Defect, erosion, and rarefaction of the villi were detected in the PBS and CotC groups, along with a significant increase in eosinophils (**Figure 1E**). But there were markedly less eosinophils in the NAP groups, and the jejunal villi in NAP-treated group were continuous, well-structured, and orderly arranged, similar to those of the naïve group (**Figure 1E**). Furthermore, there was a significant decrease in serum CAS-IgE and IgG1 levels and a significant increase of sIgA levels (**Figure 1F**, *P* < 0.01) in the NAP group. Besides, the plasma histamine and serum mMCP-1 levels of the NAP group were reduced (**Figure 1G**).

### Recombinant spores expressing NAP increase regulatory T cells (Tregs) in the spleen and intestine

**Figure 3:**
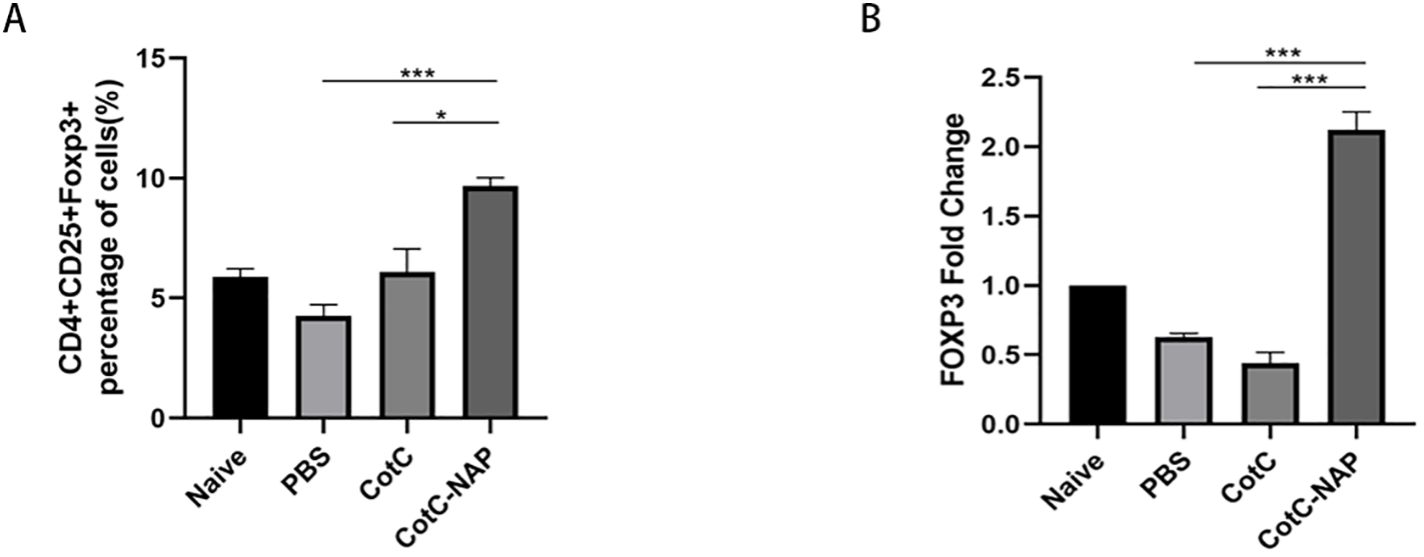
Recombinant spores expressing NAP significantly induce Tregs in the spleen and intestine. **(A)** Percentage of CD4+CD25+Foxp3+ Tregs detected by flow cytometry; 1 × 10^6^ spleen cells were stained with APC-CD25, FITC-CD4, and PE-FOXP3 antibodies. **(B)** The mRNA expression levels of Foxp3 in the jejunum detected by RT-qPCR. *P < 0.05, ***P < 0.001.

The proportions of Tregs were similar among the naïve, PBS and CotC groups (**Figure 3A**). However, the Tregs population was significantly increased in the NAP group. This effect was also confirmed by the significantly higher mRNA levels of FOXP3 in the jejunum of the NAP group compared with those of controls (**Figure 3B**).

### Recombinant spores expressing NAP skew the allergic Th2 response toward a protective Th1 response

**Figure 4:**
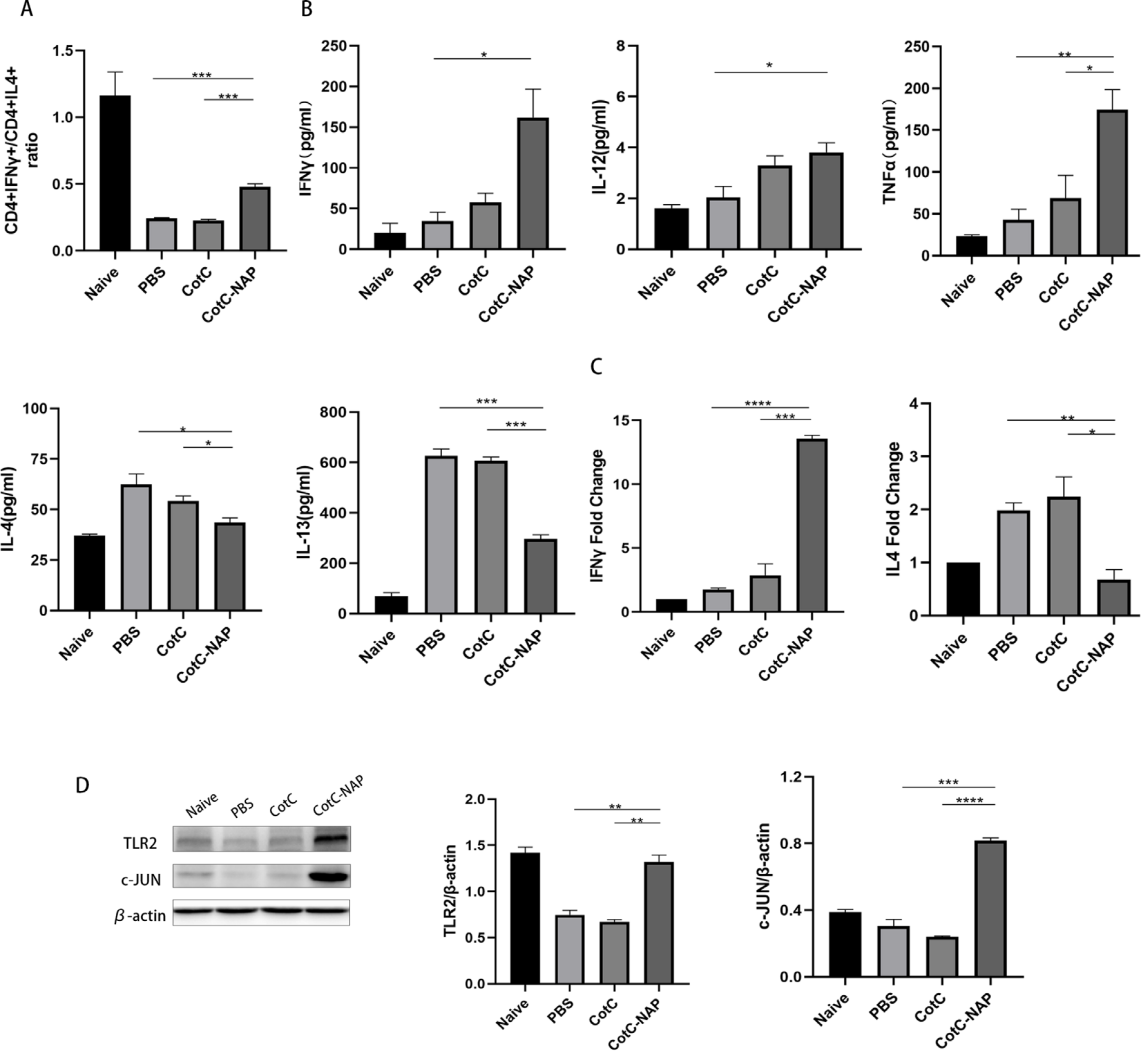
Recombinant spores expressing NAP skew the allergic Th2 response toward a protective Th1 response. **(A)** Proportions of CD4+IFNγ+ T cells and CD4+IL4+ T cells in splenocytes detected by flow cytometry; 1 × 10^6^ splenocytes were stained with FITC-CD4 and APC-IL4 or PE-IFNγ antibodies. The ratio of these two types of cells in all groups is shown. **(B)** ELISA for detecting the cytokine levels of IL-12, IFNγ, TNFα, IL-4, and IL-13 in the spleen. **(C)** RT-qPCR detection of the mRNA expression levels of Il4 and Ifng in the jejunum. **(D)** Protein levels of TLR2, p-NFκB, NFκB, c-JUN, and IL-4 in the jejunal tissue detected by western blot, quantified relative to β-actin using densitometric analysis. Data are presented as mean ± SEM. *P < 0.05, **P < 0.01, ***P < 0.001, ****P < 0.0001.

We also detected the CD4+IFN-gamma+ (Th1) cells and CD4+IL4+ (Th2) cells using flowcytometry. In the CAS allergic mouse model, the ratio of CD4+IFN-gamma+ Th1/CD4+IL4+ Th2 (approximately 0.2) was obviously decreased compared to that in the naïve group (approximately 1), reflecting the shift of Th2 immune during milk allergy. We found that the CotC-NAP *Bacillus subtilits* spores treatment restored Th1/Th2 imbalance to some extent (**Figure 4A**), compared with that of the PBS and CotC mice groups. Consistently, the splenocyte culture supernatant of the control groups had significantly higher Th2-type cytokines (IL-4 and IL-13) than the NAP group (**Figure 4B**). However, the contents of Th1-related cytokines (IL-12, IFN-γ, and TNFα) were significantly increased in the NAP group (**Figure 4B**). Similar differences among groups were detected in IFN-γ and IL-4 mRNA levels of the jejunum (**Figure 4C**). Moreover, the protein levels of TLR2 and c-JUN in the jejunum were significantly elevated in the NAP group (**Figure 4D**).

### NAP stimulates Th1 cytokines and M1 macrophage polarization through TLR2 signaling

**Figure 5:**
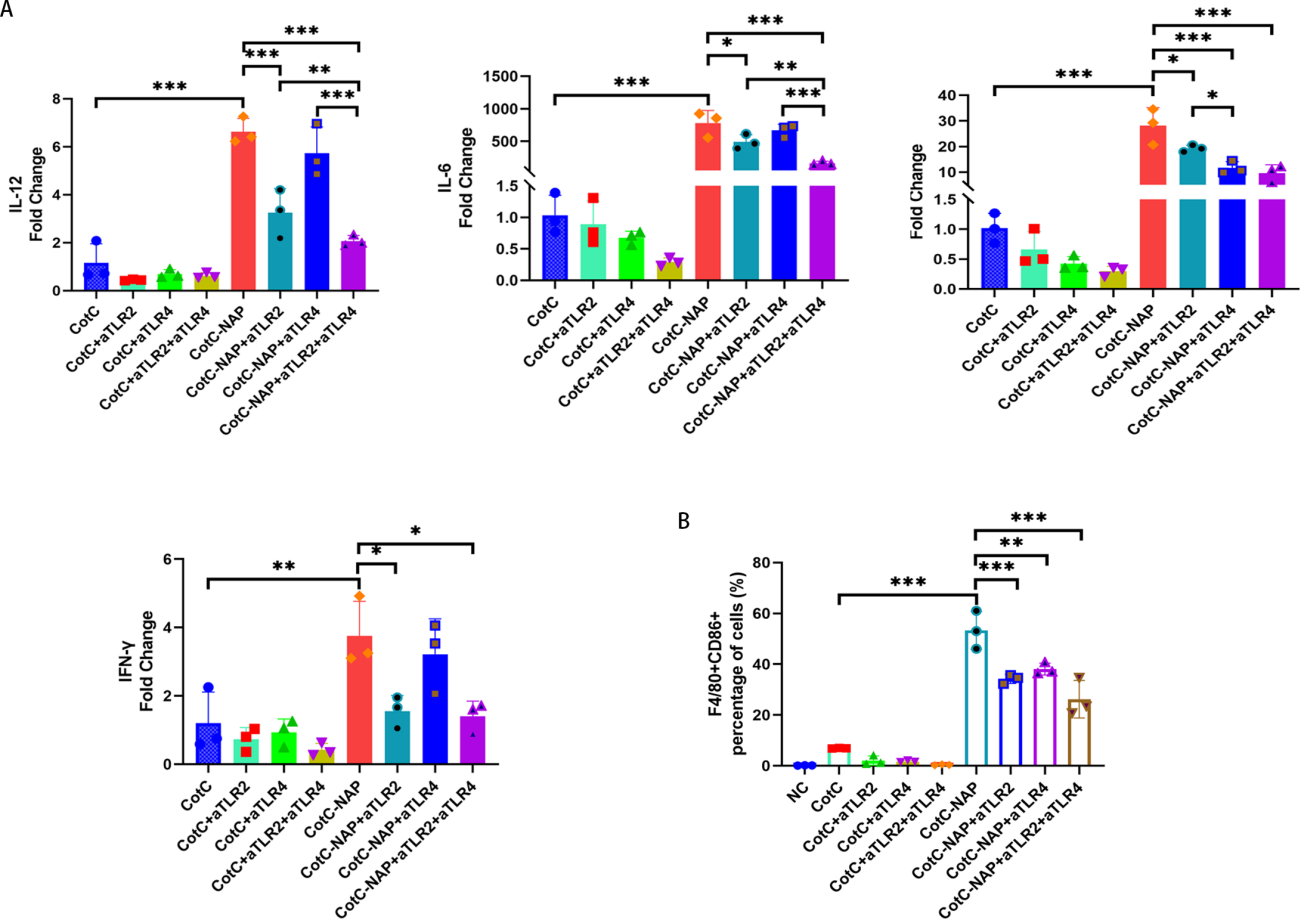
NAP stimulates Th1 cytokines and polarizes M1 macrophages through TLR2 and TLR4. **(A)** mRNA expression levels of Il12, Il6, Il1b, and Ifng measured by RT-qPCR. **(B)** Percentage of F4/80+CD86+ M1-type macrophages obtained by flow cytometry. Data are presented as mean ± SEM (n = 3/group). *P < 0.05, **P < 0.01, ***P < 0.001.

**Figure 6:**
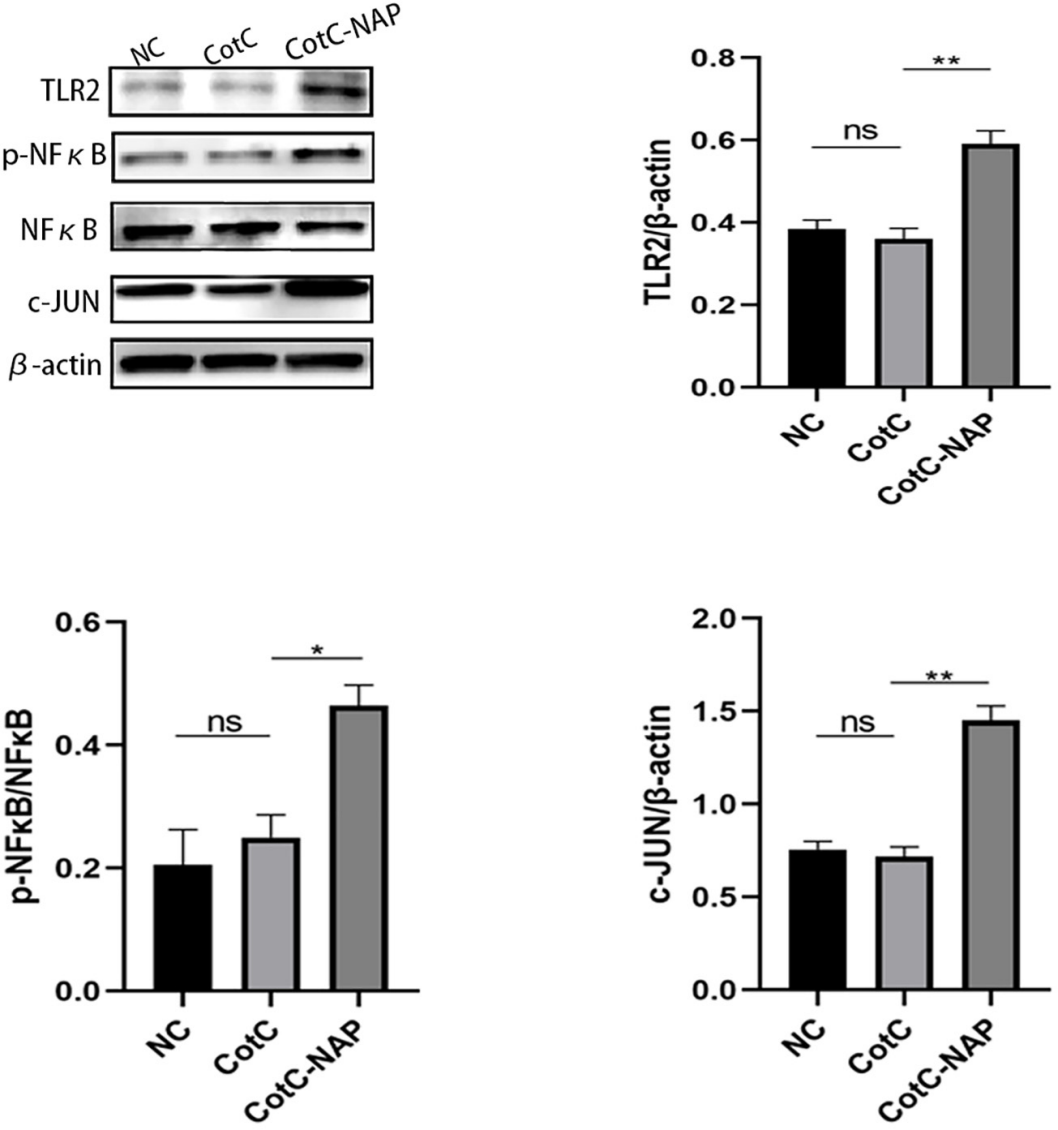
NAP enhances the expression of TLR2, p-NFκB, and c-JUN. BMDMs were stimulated with inactivated spores for 48 h and then the expression levels of TLR2, p-NFκB, NFκB, and c-JUN were detected by western blot relative to β-actin using densitometric analysis. Data are presented as mean ± SEM (n = 3/group). *P < 0.05, **P < 0.01.

Furtherly, we investigated the effect of NAP on macrophage polarization by isolating and inducing BMDM cells (**Supplementary Figure S1**). We can see from **Figure 5A** that both CotC and CotC-NAP significantly increase the expression levels of IL-1β, IL-6, IL-12, and IFNγ in BMDMs; however, the stimulatory effect of CotC-NAP was more pronounced than that of CotC. Neutralizing antibodies against both TLR2 and TLR4 significantly inhibited the induction of these cytokines by NAP, although the inhibition effect of TLR2 antibody alone was more significant than that of TLR4 antibody alone. The cytokine profile of CotC-NAP-stimulated BMDMs suggested a phenotype of M1 polarization. Flow cytometry showed that CotC-NAP and CotC significantly increased F4/80^+^CD86^+^ M1-type macrophages, although CotC-NAP was more effective than CotC (*P* < 0.001). Similarly, the neutralizing antibodies against TLR2 and TLR4 significantly inhibited the NAP-induced M1 polarization (**Figure 5B**). Western blotting showed that compared with the unstimulated control, CotC-NAP, but not CotC, significantly enhanced the expression of TLR2 and c-JUN, and the phosphorylation of NF-κB (**Figure 6**).

## Discussion

Using a mouse model, we provide evidence that *B. subtilis* spores expressing NAP can attenuate IgE-mediated CAS allergy. Additionally, we demonstrate that the mechanism involves NAP activation of the TLR2 signaling pathway.

The development of an allergy or tolerance is closely related to the intestinal mucosal barrier (30, 31). The spores of *B. subtilis* are very stable and can tolerate the strong acidic environment of the stomach, where they colonize, germinate, multiply, and reform spores in the intestine (32). We verified that *B. subtilis* survived in the intestine for up to 96 h with one dose of spores and for 21 days after six weeks of oral administration. This stability would facilitate obtaining a long-term immunosuppressive effect in the intestine of the recombinant protein NAP expressed on the spore surface.

Milk allergy, including CAS allergy, results from an overactivated Th2 immune response, characterized by a high level of antigen-specific IgE, Th2-type cytokines, and infiltration of eosinophils (33, 34). Imbalance of the Th1/Th2 immune status is decisive in the occurrence of allergic reactions (35, 36). Allergens bind with IgE and IgE receptor (FcϵRI) and then form a complex to promote mast cells and basophils to release large amounts of soluble mediators, resulting in rapid allergic reaction symptoms (37, 38). Histamine is an important marker of mast cells and basophils degranulation (39) and mMCP-1 is a chymotrypsin secreted by intestinal mucosal mast cells. Previous studies indicated elevated serum mMCP-1 levels in intestinal anaphylactic reactions, which directly correlated with the severity of anaphylaxis (40, 41). We found that NAP effectively reduced plasma histamine and serum mMCP-1 levels. This suggests inhibition of the degranulation level of mast cells in the intestinal mucosa, leading to blockage of the release of mediators required to mediate the allergic response and suppression of the onset of the allergic reaction. Furthermore, the immune function of the intestinal mucosa depends on the level of specific sIgA antibodies (42). Oral administration of NAP spores effectively induced a specific mucosal immune response in mice, prompting the production of protective sIgA antibodies in the intestinal mucosa.

The main task of Tregs is to correctly differentiate among commensal microorganisms, pathogenic microorganisms, and foreign food components and to immunize them against tolerance and an immune response, respectively (43). Moreover, Tregs in the intestine differ from those in other organs. Antigen receptors for Tregs in the intestine have a gut-specific phenotype and function to suppress immune responses to harmless antigens and commensal flora in the diet (44). Tregs in the lamina propria of the intestinal mucosa are essential in maintaining immune balance and oral tolerance, especially FOXP3^+^ Tregs (45, 46). In our previous study, we elucidated that oral administration of recombinant Bacillus subtilis spores expressing NAP could suppress peanut allergy via up-regulation of Tregs (28). Other studies also showed that IL-10 secretion from the gastric mucosa was increased in children with HP infection, and NAP inhibited Th2 responses by activating FOXP3^+^Tregs in an asthma model (47, 48). In this study, recombinant spores containing NAP significantly induced a high proportion of Tregs in the mouse spleen and intestine, which has an important role in food tolerance.

We found that NAP significantly elevated the ratio of Th1 cells (CD4^+^IFNγ^+^T cells) to Th2 cells (CD4^+^IL4^+^T cells). Further, we found high levels of IFN-γ, IL-12, and TNF-α, as well as low levels of IL-4 and IL-13 in mice orally administered with NAP spores. These data suggest that NAP is beneficial in promoting Th1 immune responses. Moreover, NAP is currently being studied in tumor therapy given its ability to induce TLR2 transcripts and promote peripheral blood mononuclear cells of cancer patients to secrete IFN-γ and IL-12 (49–53). Once TLR2 signaling is activated, the bridging protein MYD88 activates the downstream factors NF-κB and transcription activator protein 1 (AP-1) to secrete cytokines such as IL-12 (54). Additionally, the transcriptional regulator c-JUN is an important element of the JAK/STAT pathway and a component of AP-1 (55). Indeed, we found significantly increased levels of TLR2 and c-JUN expression, and significantly decreased levels of IL-4 expression treating with NAP. And we found the similar results on BMDMs. Consistently, treatment of BMDMs with NAP spores effectively induced the differentiation to M1-type macrophages, which secrete IL-12 to promote Th1-type immune responses (56). Besides, we found increased expression of IL-12, IFN-γ, IL-1β and IL-6 in BMDMs cultured with NAP spores. In addition, we found these effects was reversed by treatment with neutralizing antibody against TLR2 and/or TLR4. Although NAP appears to activate both TLR2 and TLR4-dependent signaling pathways in BMDMs, the binding of NAP is stronger for TLR2 antibody,suggestting that NAP mainly activates TLR2-dependent signaling pathways. We surprisingly found the synergism using anti- TLR2 and anti- TLR4 neutrolizing antibodies, but we did not compare the difference in TLR2 or TLR4 protein expression before and after CotC-NAP stimulation, which phenomenon deserves our future exploration in the future.

Our results revealed that the imbalance between Th1 and Th2 immune responses was a key immune mechanism in the development of allergic diseases, which provided strong evidence for the hygiene hypothesis. In addition tothe induction of Treg cells, we also found NAP could also alleviate allergic symptoms by activating Th1-type immune response in this study. However, this study have lots of limitations. Future work will focus on the molecular mechanism of NAP on M1 macrophages and will try to unveil the effects of NAP to MLN cells or macrophages in intestine in CAS-allergic mice.

Thus, we demonstrate that oral administration of NAP spores can reduce CAS-induced allergic responses, and demonstrate that NAP promotes Th1 immune responses through activation of the TLR2-dependent signaling pathway. Our data may provide a basis for future studies targeting NAP in the suppression of allergy, which may offer an important clinical approach for preventing allergic diseases.

## Data Availability

The original contributions presented in the study are included in the article/**Supplementary Materials**, further inquiries can be directed to the corresponding authors.
